# Income Changes Due to Disability Ratings and Participation in Economic Activities Caused by Industrial Accidents: A Population-Based Study of Data from the Fourth Panel Study of Workers’ Compensation Insurance (PSWCI)

**DOI:** 10.3390/ijerph15112478

**Published:** 2018-11-06

**Authors:** Suk Won Bae, Sehyun Yun, Ye Seol Lee, Jin-Ha Yoon, Jaehoon Roh, Jong-Uk Won

**Affiliations:** 1 Department of Public Health, Yonsei University, Seoul 03722, Korea; newzzanggu@hanmail.net (S.W.B.); yeseol.lee127@gmail.com (Y.S.L.); 2 The Institute for Occupational Health, Yonsei University College of Medicine, Seoul 03722, Korea; justinyun9@gmail.com (S.Y.); FLYINYOU@yuhs.ac (J.-H.Y.); JHROH@yuhs.ac (J.R.); 3 Graduate School of Public Health, Yonsei University, Seoul 03722, Korea; 4 Incheon Workers’ Health Center, Incheon 21633, Korea; 5 Institute of Health Services Research, Yonsei University College of Medicine, Seoul 03722, Korea; 6 Department of Preventive Medicine, Yonsei University College of Medicine, Seoul 03722, Korea

**Keywords:** industrial accident, disability rating, participation in economic activities, workers’ compensation insurance

## Abstract

Industrial accidents cost a huge amount of money, but they also have negative consequences in many respects. We analyzed the data of the first to fourth panel study of workers’ compensation insurance (PSWCI). Repeated measures ANOVA was used to compare the annual income before and after the industrial accident, and a general linear model was used to identify changes in income due to disability ratings and participation in economic activities. The wages before the industrial accident and the annual income varied among the disabilities ratings. In addition, for affected workers, the average income during four years post-accident was lower than the average income before the accident. Regression analysis to see changes in income after the industrial accident showed that the group with a disability rating of 11–14 and no injuries had a suffered a greater income decrease than those with a disability rating of 1–3, and the unemployment group saw a greater decrease in income than the employment group. Workers who were affected by industrial accidents received lower incomes than before the accident, and even considering different disability ratings, there was a greater decrease in income among the unemployed group than in the working group.

## 1. Introduction

According to the Korean Ministry of Employment and Labor (2017), 18.4 million workers from approximately 2.5 million businesses were eligible for the Workers’ Compensation Insurance. Among them the number of victims was 90,656, and accident rate was 0.49% [1]. This was a 0.01% decrease from that of 2015, but due to the increased number of business sites and employees, the coverage of industrial insurance continued to increase [2]. In 2015, the manufacturing industry constituted 29.12% of total work-related injuries and illnesses and the construction industry 29.54% [3]. Industrial accidents both directly and indirectly cause significant losses to the government, business owners, and employees. In 2016, direct compensation for industrial accidents was estimated to be 4 trillion KRW and when accounting for indirect losses, it amounts to about 21 trillion KRW [1].

Not only industrial accidents cost huge amount of money, but they also have negative consequences in many respects [4]. On a social level, social costs including medical fees and compensations are incurred [5,6,7,8]. Workplaces suffer from productivity loss due to absence caused by the accidents [9]. On an individual level, consequences such as disability, psychological problems, and unemployment can have negative impact on workers and their families even after industrial accidents [10,11,12,13,14].

In particular, the loss of labor capacity that follows industrial disasters could cause economic difficulties for those who are affected [15]. It is likely that disability will decrease their working ability and thus force the affected to change their roles or workplaces. As a result, there is a high chance that they will find jobs with lower wages or, even worse, lose jobs [16]. Thus, unemployment and disability caused by industrial accidents can change the lives of the industrial workers completely in terms of economic capacity and psychological consequences [17].

Research on victims of industrial accidents in relation to their return to employment reveals that gender, age, marital status, education level, type of business, occupation, workplace size, employment period and type, average wage, disability level and type, hospitalization period, recovery period, hospital characteristics, physicians, and employers are all major factors [18,19,20,21,22,23,24,25,26,27]. However, studies that directly deal with disability levels and income changes of victims of industrial accidents are insufficient.

Therefore, the present study uses data from the workers’ compensation insurance panel study to identify how the income of workers following industrial accidents changes depending on their disability grades and economic activities.

## 2. Methods

### 2.1. Study Design and Participants

We used the data from the first to fourth panel study of workers’ compensation insurance (PSWCI) organized by the Korean Workers’ Compensation and Welfare Service. The PSWCI was conducted to objectively examine the effects of the policy system created to aid industrial workers and to help provide proper medical care and assistance in their returning to economic activities. In 2012, the 82,493 workers who had completed medical care were selected as the population and stratified by gender, age, region, disability grade, and recovery service, resulting in 2000 samples. Currently, four trials of the survey have been conducted on the sample population. The surveys were conducted by computer-assisted personal interviewing (CAPI), a one-to-one and face-to-face survey guided by professional interviewers [27,28,29,30,31,32].

In this study, the analysis data were collected by referring to the PSWCI and using the following procedure. After the fourth survey, 1660 out of the initial 2000 samples were retained, but only 1588 had continuously participated in all four surveys. Of those who had participated in all four surveys, the final 1538 samples were selected excluding 50 industrial workers who had not participated in the survey or had descriptive variables that were difficult to estimate or were unobserved.

### 2.2. Sociodemographic Characteristics

Age was considered as a continuous variable and the groups were divided by five from “<30” to “≥60” in increments of 10 years. Marital status was divided into three groups: “Not Married”, “Married”, and “Others (separated, divorced, widowed, etc.)”. Education level was categorized by eight groups in the PSWCI but we re-grouped into three: “Less than high school”, “High school”, and “College or above”.

### 2.3. Occupational Characteristics

The industrial and occupational classifications were categorized according to the Korean Standard Industrial Classification (KSCI) and the Korean Standard Occupational Classification (KSCO) respectively. Based on the International Classification of Standards and Industry (ISCI), the KSCI groups the industrial and occupational classifications into three, accounting for number of cases of work-related injuries and illnesses. As a result, according to KSCI method, the industrial and occupational classifications were categorized into “Manufacturing”, “Construction”, and “Others”. The KSCO is based on the International Standard Classification of Occupations (ISCO) and the samples were categorized by “White collar”, “Service” (service sector workers including sales), and “Blue collar (including others etc.)”.

Recuperation periods were grouped into three: “≤6 months”, “7–12 months”, and “>12 months”.

There are 14 disability ratings from Grade 1 to 14 under Korea’s Industrial Accident Compensation Insurance Act, and a lower rating indicates more serious disabilities [33]. Disability ratings were categorized into five groups: “1–3” (critical), “4–7” (severe), “8–10” (moderate), “11–14” (mild), and “None”.

Participation in economic activities was divided into six groups, including returned to work, re-employment, self-employment, unpaid family worker, unemployment, and non-economic activities. Among the six groups, the first four groups—such as the return to the original job, re-employment, self-employment, and unpaid family worker—were considered “economic activity participants” while the latter two groups, including unemployment and non-economic activities, were considered “economic activity non-participants”.

For accurate analysis, those who had been employed for four consecutive years were classified as “Returned to Work” while those who had not been employed for four consecutive years were classified as the “Non-RTW”. Those who had repeatedly been employed and unemployed were classified as “Others”.

### 2.4. Main Outcome Variables

The income before the industrial accident was estimated by the answer to the question “What is the average monthly wage at the workplace (job) where the industrial accident has occurred?” This was then multiplied by 12 to calculate annual income. When the recovery period ranged from one to two years, we estimated the 2012 annual income by multiplying the 2011 pre-accident income by 1.0376, the average pay increase of all workers in 2012. When the recovery period was longer than two years, however, it was impossible to estimate the pre-accident income, which was therefore excluded. The post-accident income was estimated to be the sum of the individual’s earned income and non-work income. Non-work income included industry-related income, i.e., lump sum compensation and disability pensions, excluding property income and private income prior to the accident or unrelated to the industrial accident. The average wage increase rate reported by the Ministry of Employment and Labor was used to compare the income before and after the industrial accident and was all adjusted to fit the wages of 2015 [34].

### 2.5. Statistical Analyses

The comparison of the social and professional characteristics and the mean income before the industrial accident of the subjects was made using the *t*-test and ANOVA. Repeated measures ANOVA was used to compare the annual income before and after the industrial accident, and in addition, the average income before the accident was compared with the average income over the four-year period after the accident. A paired *t*-test and the repeated measures ANOVA were performed to compare the pre-accident income and the average income of four years after the accident with respect to the general characteristics of the subjects. A general linear model was used to identify changes in income due to disability ratings and participation in economic activities. The dependent variable was defined as the average of the first to fourth-year income excluding the income prior to the industrial accident. All analyses were completed using the SAS statistical package version 9.4 (SAS Institute, Cary, NC, USA).

## 3. Results

The general characteristics of the survey subjects are as shown in (Table 1). The average annual income before the industrial accident was 26.82 million KRW. There were more men participating in the survey than women as men composed 82.8% (1274 men) of the total survey personnel. A large proportion of participants were in their 50s. The income prior to the accidents was relatively higher for men (*p* < 0.0001). Income differences before the industrial accident were present according to age (*p* < 0.0001) and disability ratings (*p* = 0.0017).

Pre-accident income was higher in the group that participated in economic activities after the industrial accident than the group that stayed unemployed (*p* < 0.0001).

The wages before the industrial accident and the annual income varied among the disability ratings (*p* < 0.0001) (Table 2). Incomes for the first year after the industrial accident were higher than those before the accident for all workers who were affected, except those who did not suffer from any disabilities (“None”). The second-year income dropped sharply but showed a gradual increase in following years. In addition, for affected workers, the average income during four years post-accident was lower than the income before the accident (*p* = 0.0005). Income changes were also observed in the earned income and non-work income respectively (Supplementary Materials).

In the case of employed population, those with disability ratings between one and seven showed higher annual and average income for four years than income before the industrial accident (*p* < 0.0001). However, the unemployment group and others showed lower annual (*p* < 0.0001, *p* < 0.0001) and average income (*p* = 0.0004, *p* = 0.0003) (Table 3).

Table 4 shows the difference in pre-accident income and the average income over the four years after the industrial accident. The total income after the accident decreased significantly compared to the previous gross income (*p* < 0.0001), and with the exception of the age group “30–40”, the four-year average income was lower than the income before the accident. In order to identify the income changes before and after the accident, a regression analysis was performed with the dependent variable as a four-year average of post-accident income minus the income before the industrial accident and the independent variables as general characteristics, employment status, disability grade, and others (Table 5). Adjusting for all covariates, income for men showed a greater decrease after the accident than that of women (*p* = 0.0090), and manufacturing industries presented a greater decrease than the construction industry (*p* < 0.0001). Compared to the disability rating one through three group, the rest showed reduced income and there were statistically significant decreases in both the disability rating 11 through 14 group and no disability group (*p* = 0.0321, *p* = 0.0200). The unemployment group had significantly decreased income compared to those who participated in economic activities (*p* < 0.0001).

## 4. Discussion

Industrial accidents not only impair, if not completely eliminate, the worker’s work capacity but they may also cause physical and mental disabilities. This can limit labor market participation, reduce annual income, and therefore threaten household livelihoods [35,36,37]. It has long been criticized that those who are compensated due to industrial accidents through the compensation system show more unfavorable outcomes than those uncompensated [38]. This study was conducted to identify the changes in income before and after the industrial accidents of affected workers whose recovery periods had ended. The income before the industrial accident is not related to industrial accident characteristics and therefore shows similar pattern as the general income characteristics. Nevertheless, we believe that the reason work injury patients have lower pre-accident incomes than work-related illness patients is that physical injuries occur more in the service sector and blue collar jobs than in white-collar job environments. Furthermore, the reason the pre-accident income of the victims with no disabilities after an industrial accident is lower than that of those who were injured is that many of the uninjured belong to the service sector where accidents caused are usually minor. According to the 2016 analysis on the status of industrial accidents, accident rate for white-collar workers was 8.8%, the blue-collar 83.7%, and the service sector 7.3%. The death toll among the service sector and the sales sector were 0.4% and 0.2%, respectively, which is minimal [1]. Under Korea’s Industrial Accident Compensation Insurance Act, disability compensation is provided depending on the disability rating. Disability pensions are provided for Grades 1–3, or recipients can be paid half the pension amounts for the first four years in advance. Recipients for Grades 4–7 can choose between a “pension” option and “lump sum compensation” option. All recipients for Grades 8–14 are paid lump sum compensations [33]. The change in income before and after an industrial disaster was classified by disability rating to show that first-year income after the termination of medical care increased from the income prior to accident for all workers excluding the uninjured. This is believed to be related to the lump sum payment made on the day of the accident. Without the lump sum payment, the income in the second year rapidly decreases. This reduction in income can also be attributed to continued unemployment in the second year. Accordingly, the reason for gradual increase in income over time is most likely due to reemployment. We believe that the reason the income of those with no disabilities decrease after an industrial accident is because the injured cannot return to their original job or find another job even though they do not have any disabilities. In addition, the average income over four years post-accident decreased compared to the income prior to the industrial accident (Table 2). This result is similar to that of an existing research [34], and we believe that this is due to insufficient compensation or difficulties in employment after industrial accidents. We chose the four-year average income to compare with the pre-accident income since the fourth-year income reflects future income trends and the first-year income includes lump sum payment effect and thus deemed inadequate for this study. Those with an up to Grade 7 disability rating, who are eligible for the disability pension plan, showed higher annual and four-year average income than the pre-accident income. However, the unemployed, even if they were eligible for pensions, saw a decrease in both annual and four-year average income after the first year (Table 3). This means that compensation is sufficient for the employed but not for the unemployed. The Ohio Workers’ Compensation for industrial accidents is provided depending on the severity and types of wage loss and injury. In cases where wage losses make up two-thirds of total losses, the compensation varies depending on whether workers return to work; two-thirds of average weekly wage is compensated for up to 52 weeks for those returning to work and 200 weeks for those not returning [39]. American Medical Associations (AMA)’s Evaluation of Permanent Disability guide is used to determine the rating of permanent disabilities of workers from industrial accidents in California. The compensation varies depending on the employment coefficient and on the number of employees, and whether there was a legal employment offer; employees who are offered proper employment receive two-thirds of the average weekly wage minus 15% and those not offered receive two-thirds of the average weekly wage plus 15% [40]. In summary, the economic status of workers declines after industrial accidents, regardless of the degree of disability. Even those who usually have less difficulty in finding jobs—such as disability ranking 8 to 10, 11 to 14, and no disability—saw a decrease in income. There are a few limitations with this study. Since the survey was conducted at a specific period, the study does not effectively remove retrospective bias. Also, even though this was a panel survey, due to the excluded personnel, it was not weighted. Finally, the disability benefit that was given in the form of a lump sum or prepayment may have caused the first-year income to be higher than that of following years. Nevertheless, this study has strength in that it is Korea’s first panel study on workers’ compensation insurance, includes a relatively large sample, and has a sufficient tracking period to see income changes.

## 5. Conclusions

In conclusion, workers who were affected by industrial accidents received lower incomes than before the accident, and even considering the different disability ratings, there was a greater decrease in income among the unemployed group than in the working group. Therefore, efforts should be made to improve the return-to-work ratio after industrial accidents. It is also necessary to consider different disability compensation measures depending on the return-to-work status of the workers.

## Figures and Tables

**Table 1:** General characteristics of study subjects by annual income before industrial accidents status (unit: million KRW).

Variables	Total (*N* = 1538)		Income before Industrial Accident		*p*-Value *
	*N*	%	Mean	SD	
Annual income	1538	100%	26.83	12.56	
Age					<0.0001
<30	92	6.0	21.98	8.28	
30–39	219	14.2	28.35	11.44	
40–49	383	24.9	30.09	13.29	
50–59	551	35.8	27.57	12.99	
≥60	293	19.1	21.56	10.53	
Sex					<0.0001
Male	1274	82.8	29.20	12.27	
Female	264	17.2	15.37	5.86	
Marital status					<0.0001
Not married	220	14.3	23.97	8.76	
Married	1108	72.0	28.01	13.09	
Others	210	13.7	23.57	12.10	
Education level					<0.0001
Less than high school	620	40.3	24.01	11.20	
High school	677	44.0	28.33	12.68	
College or above	241	15.7	29.86	14.06	
Industry					<0.0001
Manufacturing	582	37.8	27.27	12.52	
Construction	406	26.4	31.64	11.36	
Others	550	35.8	22.81	12.14	
Occupation					<0.0001
White collar	148	9.6	31.21	15.54	
Blue collar	1273	82.8	26.96	12.14	
Service	117	7.6	19.83	9.70	
Accident type					0.0110
Injury	1413	91.9	26.54	12.27	
Disease	125	8.1	30.13	15.15	
Work period					<0.0001
<1 year	993	64.6	25.91	11.56	
1–<3 years	201	13.1	23.07	10.20	
≥3 years	344	22.4	31.67	15.04	
Recuperation period					0.0007
≤6 months	909	59.1	26.05	12.09	
7–12 months	505	32.8	27.63	13.39	
>12 months	124	8.1	29.31	12.07	
Disability rating					0.0017
1–3	6	0.4	26.63	6.96	
4–7	55	3.6	28.78	13.51	
8–10	280	18.2	27.01	11.65	
11–14	922	60.0	27.35	12.82	
None	275	17.9	24.51	12.28	
Participation in economic activity					<0.0001
Economic activity participant	1113	72.4	27.78	12.62	
Economic activity non-participant	425	27.6	24.35	12.08	

* Analyses were done by using *t*-test, ANOVA, SD: Standard Deviation.

**Table 2:** Relationship between disability rating and annual income changes before and after industrial accident (unit: million KRW).

	Income before Industrial Accident (2012) *		First (2012) *		Second (2013) *		Third (2014) *		Fourth (2015)		*p*-Value ^‡^	First to Fourth (2012–2015) ^†^		*p*-Value ^§^
	Mean	SD	Mean	SD	Mean	SD	Mean	SD	Mean	SD		Mean	SD	
Disability rating											<0.0001			0.0005
1–3 (*N* = 6)	29.93	7.82	42.78	37.85	22.16	11.74	20.01	9.08	21.21	7.85		26.54	11.83	
4–7 (*N* = 55)	32.34	15.18	52.47	32.42	22.13	19.70	23.01	16.70	26.43	16.20		31.01	14.48	
8–10 (*N* = 280)	30.35	13.09	51.56	23.54	23.03	21.44	20.18	16.42	21.46	15.65		29.06	15.42	
11–14 (*N* = 922)	30.73	14.40	33.29	19.49	24.22	18.22	23.58	16.49	24.40	15.43		26.37	15.09	
None (*N* = 275)	27.54	13.80	22.50	15.60	23.85	16.85	22.56	15.84	23.96	16.55		23.22	14.29	

* All adjusted to fit the wages of 2015. ^†^ Average of the first to fourth-year income. ^‡^ Analyses were done by using repeated measures ANOVA. ^§^ Analyses were done annual income before industrial accident and the average of the first to fourth-year annual income by using repeated measures ANOVA.

**Table 3:** Annual income characteristics between the participation of economic activities and disability rating (unit: million KRW).

Variables		Income before Industrial Accident (2012)		First (2012)		Second (2013)		Third (2014)		Fourth (2015)		*p*-Value ^§^	First to Fourth (2012–2015)		*p*-Value ^||^
		Mean	SD	Mean	SD	Mean	SD	Mean	SD	Mean	SD		Mean	SD	
RTW type	Disability rating														
Returned to work *	1–3 (*N* = 1)	24.27		25.33		36.22		37.01		36.52		<0.0001	33.77		<0.0001
	4–7 (*N* = 19)	32.47	12.09	57.20	26.99	38.08	13.93	39.67	12.75	40.02	12.83		43.74	12.17	
	8–10 (*N* = 144)	32.76	12.82	56.98	25.49	33.92	19.45	29.86	14.49	30.30	14.06		37.77	14.35	
	11–14 (*N* = 595)	32.67	14.86	36.56	20.83	31.04	16.82	29.61	15.21	29.94	13.95		31.79	14.76	
	None (*N* = 191)	29.40	14.38	24.48	16.28	28.37	15.04	27.53	14.78	28.15	14.50		27.14	13.75	
Non-RTW ^†^	1–3 (*N* = 5)	31.06	8.18	46.27	41.23	19.35	10.63	18.15	2.61	18.15	2.61	<0.0001	25.09	12.61	0.0004
	4–7 (*N* = 17)	31.81	12.01	49.14	34.27	10.69	7.98	16.14	12.51	16.14	12.51		22.10	8.48	
	8–10 (*N* = 37)	27.29	15.77	46.41	24.39	7.43	17.38	2.48	3.92	2.48	3.92		14.41	8.91	
	11–14 (*N* = 68)	24.66	16.35	25.63	17.58	3.67	7.46	2.72	4.38	2.72	4.38		8.63	6.39	
	None (*N* = 15)	21.92	8.31	19.32	13.86	4.79	10.79	1.63	4.22	1.63	4.22		6.99	5.60	
Others ^‡^	1–3 (*N* = 0)											<0.0001			0.0003
	4–7 (*N* = 19)	32.68	20.42	50.70	36.65	16.42	22.08	15.83	11.45	22.04	12.98		26.25	12.12	
	8–10 (*N* = 99)	27.99	11.78	45.59	17.95	13.03	16.69	13.15	11.72	15.68	10.86		21.86	10.11	
	11–14 (*N* = 259)	27.87	11.65	27.79	14.34	13.95	13.97	15.25	12.72	17.36	12.34		18.59	9.46	
	None (*N* = 69)	23.61	11.99	17.71	12.82	15.49	16.71	13.24	12.06	17.21	17.53		15.91	11.54	

* A four-year status of economic activity (return to the original job, re-employment, self-employment, and unpaid family worker). ^†^ A four-year status of non-economic activity (unemployment and non-economic activity). ^‡^ Status of economic activities and non-economic activities. ^§^ Analyses were done by using repeated measures ANOVA. ^||^ Analyses were done annual income before industrial accident and the average of the first to fourth-year annual income by using repeated measures ANOVA.

**Table 4:** Comparison of the income before industrial accident and the four-year average income after the industrial accident according to general characteristics (unit: million KRW).

Variables	Income before Industrial Accident *		Average of the First to Fourth-Year Income *		Difference Income from Pre to Post Accident	*p*-Value ^†^
	Mean	SD	Mean	SD	%	
Annual income	30.15	14.11	26.46	15.09	−14%	<0.0001 ^‡^
Age						<0.0001
<30	23.12	8.82	19.93	11.79	−16%	
30–39	30.07	12.28	31.65	15.38	5%	
40–49	33.43	14.44	31.21	15.51	−7%	
50–59	31.74	14.27	27.87	15.34	−14%	
≥60	26.49	13.71	19.99	11.88	−33%	
Sex						<0.0001
Male	32.81	13.79	28.99	15.01	−13%	
Female	17.27	6.59	14.28	7.79	−21%	
Marital status						<0.0001
Not married	26.82	9.89	23.15	11.15	−16%	
Married	31.50	14.67	28.36	16.05	−11%	
Others	26.58	13.37	20.33	10.56	−31%	
Education level						<0.0001
Less than high school	26.98	12.59	20.85	11.71	−29%	
High school	31.85	14.26	29.39	15.28	−8%	
College or above	33.42	15.77	32.53	17.40	−3%	
Industry						<0.0001
Manufacturing	30.64	14.07	29.30	16.47	−5%	
Construction	35.55	12.76	26.54	12.47	−34%	
Others	25.63	13.64	23.40	14.76	−10%	
Occupation						<0.0001
White collar	35.06	17.46	34.48	17.23	−2%	
Blue collar	30.30	13.64	26.19	14.72	−16%	
Service	22.28	10.90	19.34	11.50	−15%	
Accident type						<0.0001
Injury	29.82	13.79	25.91	14.55	−15%	
Disease	33.85	17.03	32.69	19.26	−4%	
Work period						<0.0001
<1 year	29.12	12.98	23.42	12.32	−24%	
1–<3 years	25.92	11.46	24.74	13.02	−5%	
≥3 years	35.58	16.90	36.24	18.95	2%	
Recuperation period						0.0618
≤6 months	29.27	13.58	26.12	14.38	−12%	
7–12 months	31.04	15.04	26.80	16.16	−16%	
>12 months	32.93	13.57	27.58	15.73	−19%	
Disability rating						0.0005
1–3	29.93	7.82	26.54	11.83	−13%	
4–7	32.34	15.18	31.01	14.48	−4%	
8–10	30.35	13.09	29.06	15.42	−4%	
11–14	30.73	14.40	26.37	15.09	−17%	
None	27.54	13.80	23.22	14.29	−19%	
Participation in economic activity						<0.0001
Economic activity participant	30.87	14.09	28.61	14.86	−8%	
Economic activity non-participant	26.61	13.74	15.96	11.39	−67%	

* All adjusted to fit the wages of 2015. ^†^ Analyses were done by using repeated measures ANOVA. ^‡^ Analyses were done by using paired *t*-test.

**Table 5:** Relationship between the general characteristics and four-year average of post-accident income minus the income before the industrial accident (unit: million KRW).

Variables	*ß* *	SE	*p*-Value
Age			
<30	ref		
30–39	1.66	1.85	0.3693
40–49	−2.10	1.83	0.2518
50–59	−3.32	1.86	0.0747
≥60	−3.60	1.93	0.0621
Sex			
Male	−2.15	0.82	0.0090
Female	ref		
Marital status			
Not married	−1.46	1.16	0.2102
Married	1.20	0.78	0.1231
Others	ref		
Education level			
Less than high school	−1.56	0.98	0.1117
High school	0.01	0.84	0.9884
College or above	ref		
Industry			
Manufacturing	ref		
Construction	−4.26	0.76	<0.0001
Others	0.26	0.67	0.7026
Occupation			
White collar	ref		
Blue collar	0.28	0.99	0.7749
Service	−0.61	1.38	0.6570
Accident type			
Injury	−0.38	1.02	0.7129
Disease	ref		
Work period			
<1 year	−3.91	0.74	<0.0001
1–<3 years	−2.17	0.95	0.0222
≥3 years	ref		
Recuperation period			
≤6 months	ref		
7–12 months	−0.81	0.60	0.1794
>12 months	−2.19	1.08	0.0428
Disability rating			
1–3	ref		
4–7	−3.40	4.50	0.4503
8–10	−4.79	4.37	0.2738
11–14	−9.34	4.35	0.0321
None	−10.24	4.40	0.0200
Participation in economic activity			
Economic activity participant	ref		
Economic activity non-participant	−7.65	0.75	<0.0001

* Statistical estimated from a general linear model that adjusted for all other covariates excluding an interesting variant, SE: Standard Error.

## Supplementary Materials

The following are available online at http://www.mdpi.com/1660-4601/15/11/2478/s1, Table S1: Relationship between disability rating and total earned annual income changes before and after industrial accident, Table S2: Relationship between disability rating and total non-work annual income changes before and after industrial accident.
