# Supplementary material for: Income Changes Due to Disability Ratings and Participation in Economic Activities Caused by Industrial Accidents: A Population-Based Study of Data from the Fourth Panel Study of Workers’ Compensation Insurance (PSWCI)
Source: Int J Environ Res Public Health. 2018 Nov 6;15(11):2478. doi: 10.3390/ijerph15112478 (PMC6266137; doi:10.3390/ijerph15112478)
Supplement: Supplementary file 1 [file ijerph-15-02478-s001.pdf]

International Journal of Environmental Research and Public Health

MDPI

Table S1. Relationship between disability rating and total earned annual income changes before and after industrial accident (unit: million KRW).

	Income before Industrial Accident (2012) *		1st (2012) *		2nd (2013) *		3rd (2014) *		4th (2015)		*p*-Value ‡	1st–4th (2012–2015) †		*p*-Value §
	**Mean**	**SD**	**Mean**	**SD**	**Mean**	**SD**	**Mean**	**SD**	**Mean**	**SD**		**Mean**	**SD**	
Disability rating											<0.0001			0.0373
1–3 (*N* = 6)	29.93	7.82	2.06	5.05	3.22	7.89	3.09	7.57	3.00	7.35		2.84	6.96	
4–7 (*N* = 55)	32.34	15.18	8.47	11.53	12.99	15.44	13.66	14.72	15.26	14.17		12.60	12.13	
8–10 (*N* = 280)	30.35	13.09	10.44	12.88	18.83	18.37	19.29	16.72	20.33	16.19		17.22	13.73	
11–14 (*N* = 922)	30.73	14.40	13.27	13.60	22.05	17.43	22.77	16.80	23.56	15.96		20.41	14.17	
None (*N* = 275)	27.54	13.80	17.11	14.71	22.31	15.61	21.84	15.76	22.96	16.23		21.05	14.04	

* All adjusted to fit the wages of 2015. † Average of the first to fourth-year income. ‡ Analyses were done by using Repeated measures ANOVA. § Analyses were done annual income before industrial accident and the average of the first to fourth-year annual income by using Repeated measures ANOVA.

Table S2. Relationship between disability rating and total non-work annual income changes before and after industrial accident (unit: million KRW).

	Income before Industrial Accident (2012) *		1st (2012) *		2nd (2013) *		3rd (2014) *		4th (2015)		*p*-Value ‡	1st–4th (2012-2015) †		*p*-Value §
	**Mean**	**SD**	**Mean**	**SD**	**Mean**	**SD**	**Mean**	**SD**	**Mean**	**SD**		Mean	SD	
Disability rating											<0.0001			<0.0001
1–3 (*N* = 6)	29.93	7.82	40.72	39.30	18.94	9.56	16.92	3.69	18.21	2.34		23.70	11.79	
4–7 (*N* = 55)	32.34	15.18	44.00	34.11	9.14	9.32	9.35	9.32	11.17	10.55		18.41	8.48	
8–10 (*N* = 280)	30.35	13.09	41.12	19.62	4.20	11.24	0.89	2.36	1.12	2.80		11.83	5.74	
11–14 (*N* = 922)	30.73	14.40	20.02	13.87	2.17	5.45	0.80	3.10	0.84	2.61		5.96	4.16	
None (*N* = 275)	27.54	13.80	5.39	6.91	1.55	8.22	0.73	2.65	1.00	4.50		2.17	4.07	

* All adjusted to fit the wages of 2015. † Average of the first to fourth-year income. ‡ Analyses were done by using Repeated measures ANOVA. § Analyses were done annual income before industrial accident and the average of the first to fourth-year annual income by using Repeated measures ANOVA.
